# Direct drive with the argon fluoride laser as a path to high fusion gain with sub-megajoule laser energy

**DOI:** 10.1098/rsta.2020.0031

**Published:** 2020-10-12

**Authors:** S. P. Obenschain, A. J. Schmitt, J. W. Bates, M. F. Wolford, M. C. Myers, M. W. McGeoch, M. Karasik, J. L. Weaver

**Affiliations:** 1 Laser Plasma Branch, Plasma Physics Division, U.S. Naval Research Laboratory, Washington, DC 20375 USA; 2 PLEX LLC, Fall River, MA 02723 USA

**Keywords:** laser fusion, argon fluoride laser, inertial fusion energy

## Abstract

Argon fluoride (ArF) is currently the shortest wavelength laser that can credibly scale to the energy and power required for high gain inertial fusion. ArF's deep ultraviolet light and capability to provide much wider bandwidth than other contemporary inertial confinement fusion (ICF) laser drivers would drastically improve the laser target coupling efficiency and enable substantially higher pressures to drive an implosion. Our radiation hydrodynamics simulations indicate gains greater than 100 are feasible with a sub-megajoule ArF driver. Our laser kinetics simulations indicate that the electron beam-pumped ArF laser can have intrinsic efficiencies of more than 16%, versus about 12% for the next most efficient krypton fluoride excimer laser. We expect at least 10% ‘wall plug' efficiency for delivering ArF light to target should be achievable using solid-state pulsed power and efficient electron beam transport to the laser gas that was demonstrated with the U.S. Naval Research Laboratory's Electra facility. These advantages could enable the development of modest size and lower cost fusion power plant modules. This would drastically change the present view on inertial fusion energy as being too expensive and the power plant size too large.

This article is part of a discussion meeting issue ‘Prospects for high gain inertial fusion energy (part 1)'.

## 1. Introduction

The superior laser target coupling with argon fluoride (ArF)'s deep UV light (*λ *= 193 nm) could enable the high target gains needed for inertial fusion energy (IFE) at much lower laser energies than previously thought feasible. The combination of deep UV light and broad native bandwidth (5 to 10 THz) would suppress laser plasma instabilities that limit the laser intensity and ablation pressures of current 351 nm frequency-tripled Nd : glass lasers which are the traditional laser drivers for fusion. ArF is thus a potentially disruptive technology for laser fusion that shares many technologies with the krypton fluoride (KrF) laser (248 nm) used on the Nike laser system at the U.S. Naval Research Laboratory (NRL) [1]. The ArF laser would use similar electron beam pumping to that used for large KrF amplifiers. It would also be able to use the ISI (induced spatial incoherence) beam smoothing technology demonstrated on Nike that enables very uniform illumination of directly driven targets and provides the capability to ‘zoom' the focal profile down to follow an imploding target [2] and thereby maintain high absorption efficiency throughout a direct-drive implosion. The KrF technology was chosen for the Nike facility because of numerous advantages for achieving laser fusion. ArF laser light in turn would be superior to KrF. Laser kinetics simulations indicate that ArF would have as much as 1.5 times higher laser intrinsic efficiency than KrF and thereby enable wall plug efficiencies of at least 10%. These advantages could enable the development of modest size and lower cost inertial fusion power plant modules that operate at laser energies well below 1 MJ.

## 2. Laser target interaction with an argon fluoride driver

**Figure 1.**
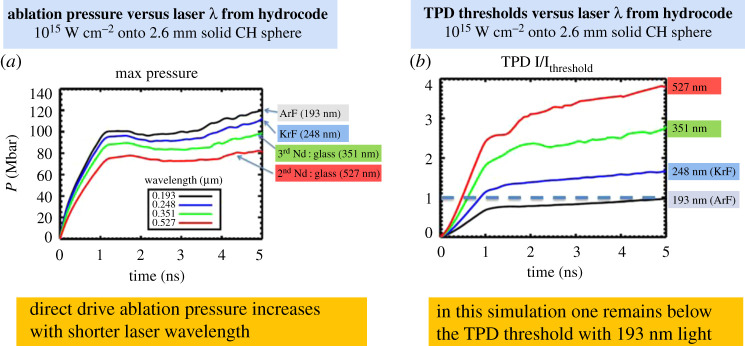
(*a*) Hydrocode-computed ablation pressures produced by 527 nm, 351 nm, 248 nm and 193 nm laser light incident onto a 2.6 mm diameter solid plastic target with vacuum intensity of 10^15^ W cm^−2^ at the spherical target's surface. The laser pulse has a 1 ns linear ramp rise followed by constant power. The pressure monotonically increases with shorter laser wavelength. (*b*) The ratio of the incident laser intensity at quarter critical density to the two plasmon decay instability threshold. This parameter monotonically decreases with shorter wavelength. (Online version in colour.)

ArF's deep UV light and expected capability to provide wide spectral bandwidth would fundamentally improve laser target coupling efficiency and allow much higher ablation pressures to be applied before one encounters unacceptable laser plasma instability (LPI). In this section, we quantify the effects as predicted by simulation codes. Figure 1*a* shows the time evolution of the ablation pressure applied to a 2.6 mm diameter solid plastic sphere from a one-dimensional simulation using the FASTRAD3D radiation hydrocode [3] for four laser wavelengths ranging from 527 nm (frequency-doubled Nd : glass) to 193 nm. The laser rays in the one-dimensional simulation are incident in parallel with an intensity distribution given by a fifth-order super-Gaussian and an initial radius of approximately 95% of the initial pellet radius, and are refractively traced through the spherical plasma. The thermal conduction is treated with a flux-limited Spitzer formulation with a flux limiter of 0.06. In the absence of an absorbing plasma, the intensity would be 10^15^ W cm^−2^ at the target's surface. The ablation pressure monotonically increases with shorter laser wavelength. The shorter wavelength is absorbed at higher plasma density, resulting in a higher density, cooler, more absorptive blow-off plasma, which results in higher ablation pressure for a given incident laser intensity. Intensity moreover is limited by LPI, and LPI thresholds increase for shorter wavelengths. Laser plasma instabilities can prevent high-performance fusion implosions by scattering the laser light and by producing non-thermal hot electrons that preheat the fuel within the imploding shell too much. The LPI intensity thresholds for given plasma conditions are higher with a shorter wavelength laser. The threshold for stimulated Raman backscatter (SRS), stimulated brillouin backscatter (SBS), and the two plasmon decay instability (TPD) all vary inversely with the laser wavelength. See for example the appendix of useful LPI formulae in reference [4]. The LPI thresholds are dependent on the plasma parameters and spatial gradients, so one must use the analytic LPI thresholds in concert with hydrocode simulations to evaluate the net effect of wavelength on laser plasma instability. As an example, figure 1*b* shows the number of times one is above the TPD instability threshold [5] for the four wavelengths and the same conditions as figure 1*a*. That parameter varies in time due to changes in the plasma density profile and temperature. The 351 nm light is well above threshold after the plasma forms. With 193 nm light, the parameter remains below 1 for these conditions and one expects little or no TPD. The plasma waves produced by TPD can accelerate electrons from the thermal distribution to speeds that allow them to pass through the imploding pellet shell and potentially cause unacceptable preheat of the deuterium-tritium (DT) fuel. SRS can also produce hot electrons, and planar laser plasma interaction experiments at 351 nm on the National Ignition Facility (NIF) indicate that with the longer scale length plasmas produced at high laser energy SRS may be a more significant source of hot electrons than TPD [6]. With ArF's 193 nm light, the plasma density scale lengths are substantially shorter than with 351 nm, which suppresses both TPD and SRS, so the relative importance of the two instabilities may be different from that encountered with 351 nm light.

Table 1 lists parameters from the one-dimensional hydrocode simulations averaged over the 2 to 3 ns interval where a long scale length coronal plasma has formed. The pressure with 193 nm light is 20% higher than with 351 nm light. The mass ablation rate is 23% higher with 193 nm light compared to 351 nm light. The higher mass ablation rate produces a cooler denser blow-off plasma ‘rocket' exhaust that can more efficiently accelerate a pellet shell to the velocities (300–400 km s^−1^) needed for an inertial fusion implosion [7]. The formula used for the TPD threshold is (230 × (351 nm/*λ*_nm_) × T_e(kev)_/L_d(µm)_) × 10^14^ W cm^−2^ where T_e_ is the electron temperature and L_d_ is the density scale length at quarter critical density. The electron temperature and density gradient scale lengths are provided in the table along with the computed TPD threshold. The laser intensity at quarter critical density is also provided. Absorption of laser light at plasma density below critical reduces the laser light intensity at critical density. The lower temperature plasma produced by 193 nm light is more absorptive than that produced by 351 nm light, and, in this case, the intensity is 86% of that computed for 351 nm light at quarter critical density. The intensity at quarter critical density is below the TPD threshold for 193 nm light while it is 2.49 times the threshold for 351 nm light. The onset of TPD with ArF light is suppressed by the combination of shorter density gradient scale length, its short wavelength and reduced light reaching critical density.

**Table 1.** The table lists computed parameters averaged over the interval from 2 to 3 ns from the simulations shown in figure 1. The electron temperature, density gradient scale length and the laser intensity at quarter critical density are listed, along with the ratio of the laser intensity at quarter critical to the TPD threshold. Absorption of the laser light before it reaches quarter critical density helps mitigate TPD. In addition, the maximum laser bandwidths demonstrated with 351 nm and 248 nm ICF laser facilities, and the projection for a 193 nm ArF system are provided. The TPD I/I_th_ ratio only includes the effect of laser wavelength. Multi-THz bandwidth could further reduce growth of TPD.

wavelength (nm)	pressure (Mbar)	mass ablation rate (gm cm^−2^ s^−1^)	Te (keV)	Ld (µm)	I @ 0.25nc (×10^14^ W cm^−2^)	TPD I/I_th_	laser bandwidth (THz)
351	84	1.82 × 10^5^	3.88	448	5.19	2.49	1
248	93	2.04 × 10^5^	3.32	318	5.11	1.45	3
193	100	2.23 × 10^5^	2.87	221	4.44	0.79	10

Laser beam smoothing using spatial and temporal incoherence with bandwidths near 1 THz have proven effective at preventing filamentation of the laser light in the coronal plasma [8] and thereby reduced other LPI that was enhanced by filamentation [9]. Still broader bandwidth laser light (multi-THz) is predicted to disrupt the coherent laser plasma wave interactions that drive LPI and may thereby directly inhibit growth of LPI [10]. The last column in table 1 lists the demonstrated bandwidths at 351 nm on the OMEGA facility [11], at 248 nm on the Nike facility [1] and the much higher predicted bandwidth available with an ArF driver from kinetics simulations. While bandwidths approaching 1 THz have been effective at mitigating filamentation, they have been ineffective at directly reducing other LPI. However, simulations and theory indicate multi-THz bandwidths may be effective at suppressing certain types of LPI. For example, laser plasma interaction simulations indicate that 2 THz bandwidth begins to suppress cross beam energy transfer (CBET), and 5 THz almost completely suppresses it [12]. CBET has been shown to be a major loss (30%) of drive power in OMEGA direct-drive implosions [13]. The 10 THz bandwidth available with ArF should therefore be more than sufficient to suppress CBET. Other simulations indicate that broad bandwidths might also suppress the growth of absolute TPD and SRS instability [14]. To obtain similar bandwidths with frequency-tripled ND : glass lasers would require addition of bandwidth enhancing systems to the laser output such as stimulated rotational Raman scattering in a diatomic gas [15,16] or optical parametric amplifiers [17]. Experimental testing of the net effects of broad bandwidth laser light on LPI awaits availability of high-energy broad bandwidth ICF lasers. Beam smoothing techniques using spatial and temporal incoherence are instantaneously highly non-uniform and can imprint non-uniformity onto a target that seeds hydrodynamic instability [18]. The rate at which the laser profile is smoothed (temporal averaging) is directly proportional to the square root of the bandwidth. Broad laser bandwidth thus has the additional benefit of reducing this imprint.

**Figure 2.**
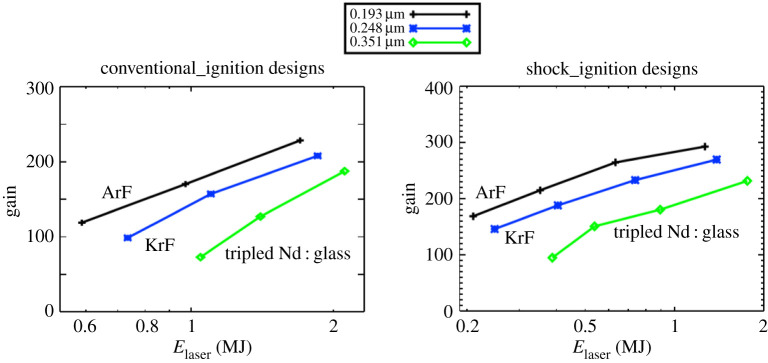
One-dimensional hydrocode simulations of target energy gains as a function of laser energy for conventional and shock-ignited targets. The simulations assumed that the laser focal diameter was zoomed down once for the conventional designs and twice for the shock-ignited designs to improve the coupling efficiency. The simulations show the advantage of using ArF's 193 nm light in comparison to 248 nm and 351 nm. (Online version in colour.)

We briefly present here the beneficial effects of using ArF laser light to achieve high-energy gain at reduced laser energy. Figure 2 shows one-dimensional hydrocode simulations of conventional and shock-ignited direct-drive implosions [19,20] as a function of laser energy and wavelength. These simulations indicate the potential yields in the absence of hydro and laser plasma instabilities. In both designs the pellets consisted of DT-loaded CH (plastic) foam ablators with radius-to-initial thickness aspect ratios of 3.74. For the given conditions, the ArF light provides higher target gains than the KrF or frequency-tripled Nd : glass options. This is due to the ArF laser light being absorbed at higher plasma density, which for a given intensity produces a higher ablation pressure and a concomitant higher hydro-efficiency. A figure of merit for the IFE application is the product of the laser efficiency times the pellet fusion energy gain. One needs this product to be at least 10 to ensure that most of the generated power is available for the grid and not taken up to power the laser system and the plant's auxiliary equipment. With a 10% (wall plug) efficient ArF laser system a gain of 100 is sufficient. The one-dimensional simulations show this to occur well below 1 MJ for the conventional designs and well below 0.5 MJ for the shock-ignited designs.

**Figure 3.**
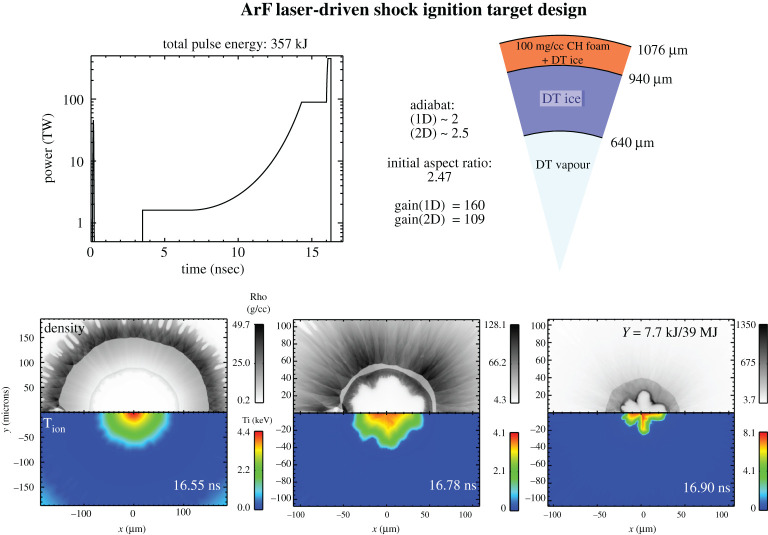
High-resolution two-dimensional simulation of a 357-kJ ArF directly driven shock-ignited target implosion with a computed yield of 37 MJ. The first frame is 550 ps after the start of the ignitor pulse. At the last frame, the target yield has reached 7.7 kJ out of an eventual 39 MJ. The top of each frame provides the mass density distribution while the bottom provides the temperature distribution. The ignition spike at the end of the pulse is 300 ps FWHM with peak power of 450 TW and energy of 135 kJ (37.8% of the total energy). This target has a lower initial aspect ratio (2.47) than the targets shown in figure 2 to improve hydro stability. This comes at the expense of somewhat lower one-dimensional gain than the 3.74 aspect ratio targets shown in figure 2. (Online version in colour.)

Suppression of LPI by ArF's combination of broad bandwidth and short wavelength would allow use of higher drive pressures for inertial fusion implosions than is feasible with current modest bandwidth 351 nm laser drivers. The higher pressure would allow one to use smaller radius lower aspect ratio targets for direct-drive implosions. Smaller radius lower aspect ratio targets are less susceptible to hydro instability and require less precision in the target fabrication and drive uniformity than higher aspect ratio targets. We provide in figure 3 a sample two-dimensional high-resolution FASTRAD3D hydrocode simulation of a directly driven shock-ignited target using a 357 kJ ArF driver where the initial aspect ratio is 2.47. The simulated two-dimensional yield is 37 MJ with a gain of 109. The two-dimensional gain was 70% of an optimized one-dimensional simulation due to the effects of laser and target nonuniformity. The simulation used the following parameters:

— 2-THz bandwidth ISI with 100 randomly polarized overlapped beams
— 1.0 µm RMS Inner DT ice perturbation
— 0.37 µm RMS outer surface perturbation in the 0.33 gm cc^−1^ foam/DT ablator – equivalent to about 0.113 µm perturbation in CH density of 1.07 gm cc^−1^.

The spectrum of the inner/outer perturbations is given by the ‘NIF spectrum' as described in Schmitt *et al*. [21], and references 18 and 19 of that paper. The high drive pressure with ArF enabled use of low initial aspect ratio (target radius/ thickness) which was found to be essential to obtain good performance in the high-resolution two-dimensional hydro simulations. Higher aspect ratio targets were found to be more sensitive to effects of the nonlinear evolution of low mode perturbations at times near stagnation.

## 3. High-energy argon fluoride science and technology

**Figure 4.**
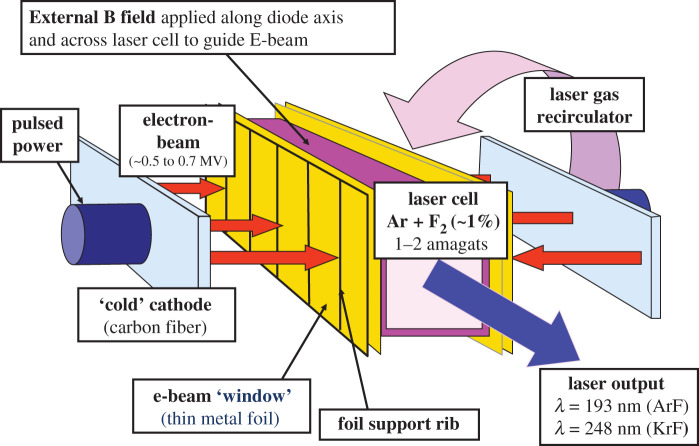
Schematic of the components of an electron beam-pumped excimer laser amplifier, including pulsed power, cathode, e-beam window, laser cell and external magnetic field to guide the electron beam into the gas cell. A laser gas recirculator can cool the laser gas and enable high-repetition rate pulsed operation (e.g. 10 pulses per second). (Online version in colour.)

The key components of an electron beam-pumped excimer laser are shown in figure 4. A pulsed power system delivers a high-current high-voltage pulse (500–800 kV) to a cathode which emits an electron beam. For the case shown, the electron beam is guided by an external magnetic field through a thin metal foil into the laser gas. The NRL Nike facility uses 20 cm and 60 cm aperture KrF amplifiers [1]. Nike has demonstrated up to 4 kJ KrF laser output. A transit time instability was observed in the 60 cm amplifier's high-current electron beam which caused undesired heating of the beam and reduced the efficiency of the electron beam deposition into the laser gas. Specialized cathodes were developed that suppress the instability so there is no physics limit to the size and current of larger systems [22]. The NRL 30 cm aperture Electra facility was built to develop and demonstrate high-repetition rate operation of a KrF electron beam-pumped amplfier. The Electra amplifier demonstrated up to 750 J output at 5 pulses per second and up to 75% electron beam transport efficiency into the laser gas [23]. The higher voltages required for larger aperture systems would reduce losses in the metal foil separating the electron-beam diode from the laser gas and enable more efficient (80%) transport into the laser gas.

While similar in many ways, there are three fundamental differences between KrF and ArF laser amplifiers. (i) An ArF gas mixture has less stopping power than a typical KrF gas mixture consisting of a 10% to 30% partial pressure of Kr. Ar has approximately half the stopping power as Kr. For a given pressure and laser aperture this requires that one use a higher current lower voltage electron beam to achieve a given deposition rate (W cm^−3^) into the gas with ArF. The Nike 60 cm amplifier uses a modest 10% Kr mixture, so this would be a modest change in parameters needed for ArF operation. (ii) At a given electron beam pump rate, ArF has lower gain than KrF, but a higher saturation fluence. (iii) There is much less absorption by F_2_ at 193 nm than at 248 nm. The predicted net effect of the above is that ArF can be substantially more efficient than KrF, but the details of an optimized ArF amplifier design and staging will differ from that for KrF. The NRL ArF programme is seeking to advance the physics understanding of the ArF laser and to develop the technological modifications needed to accommodate the differences between ArF and KrF.

**Figure 5.**
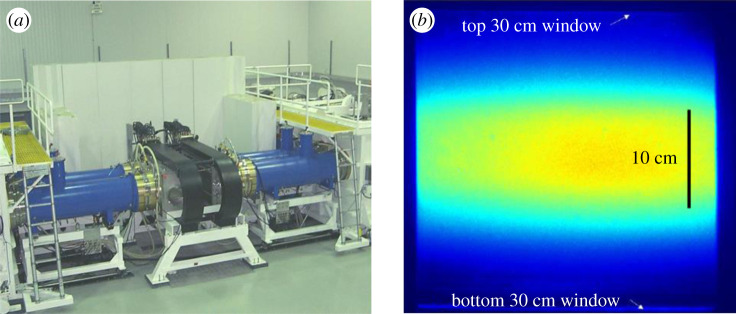
(*a*) Photo of the Electra 30-cm aperture electron beam-pumped amplifier before the x-ray shielding was installed. Two coaxial transmission lines using a water dielectric on each side of the laser cell provide a 500 kV pulse to the cathodes. (*b*) The vertical dimension of Electra's cathode was reduced from 30 cm to 10 cm to provide higher e-beam pump intensity for ArF operation. The photo shows a time resolved image of the emitted light produced by the reduced size electron beams interacting with the laser gas looking along the laser axis. The gate is 10 ns and is taken at the time of peak current. The vertical black line is shown for scale. (Online version in colour.)

The NRL Electra electron beam-pumped amplifier (figure 5*a*) has been converted from KrF to ArF operation [24]. The vertical aperture of the cathode was reduced from 30 cm to 10 cm to provide a more intense electron beam and enable pump rates above 1 MW cm^−3^ into the laser gas (figure 5*b*). Electra recently demonstrated 200 J ArF output in oscillator mode. This ArF laser energy is higher than that previously reported in publications: 95 J at Keio University Japan [25] and 92 J at Sandia [26]. Much higher energies are expected from Electra when the system is modified to a more optimized configuration for ArF operation. Measurements of the small signal gain, non-saturable absorption and saturation intensity of ArF as a function of laser gas pressure were made in electron beam-pumped amplifier experiments of both single pass and double pass configuration in a wide range of conditions. For example, at a pressure of 0.82 atm (12 psia), laser gas composition of 0.3% fluorine and 99.7% argon, and power deposition of 1.09 MW cc^−1^ the measurements yielded 3.21%/cm small signal gain, 0.16%/cm non-saturable absorption and 10 MW cm^−2^ saturation intensity [24]. Initial small signal gain measurements at temporal peak power deposition [24] as well as initial oscillator peak power laser efficiency measurements [27] are consistent with laser intrinsic efficiency of 16%. Intrinsic efficiency is defined as the ratio of laser power output divided by the electron beam power deposited in the gas for the case of continuous laser extraction. This effort is supported by NRL's 6.1 basic research programme.

High-energy pulsed ArF lasers will require relatively large transmissive and reflective optics that can operate reliably at fluences of a few J cm^−2^. We have observed no optical damage in Electra's optics after hundreds of high-energy shots at fluxes up to about 2 J cm^−2^. Much work has been done in the lithographic industry to develop ArF optics that can survive billions of shots. Researchers at Nikon have reported that their ArF grade calcium fluoride windows survive up to 20 J cm^−2^ in 20 ns pulses without bulk damage [28]. High damage threshold (29.8 J cm^−2^) fluorine-resistant coatings have been developed for KrF amplifier windows [29] that may be adaptable to ArF. While there will certainly be need to advance ArF optic technology to accommodate large components and fluences needed for ArF IFE systems, the present status indicates that such optics can be developed.

**Figure 6.**
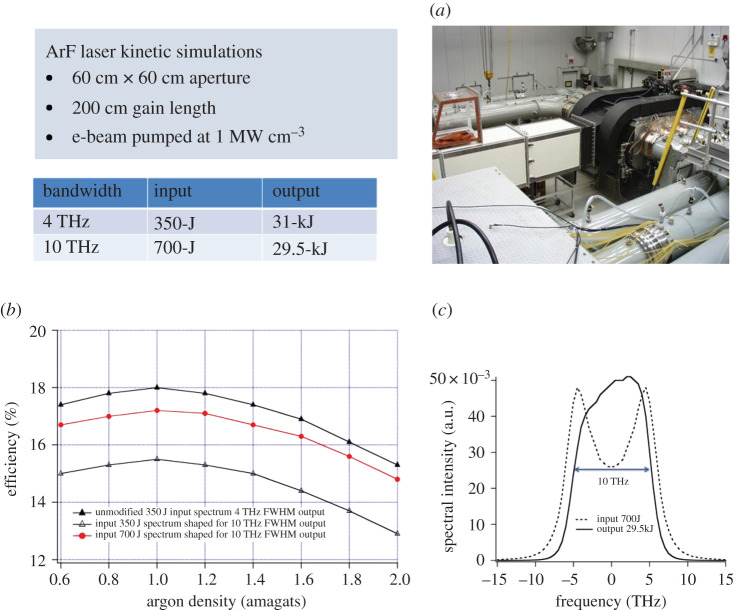
Kinetics code simulations of ArF output from an electron beam-pumped amplifier with the same aperture and gain length as used on the Nike KrF facility pumped at 1 MW cm^−3^ for 240 ns. The table provides the input and output energy for two bandwidths. (*a*) Photo of the Nike 60-cm aperture KrF amplifier. (*b*) Intrinsic efficiency versus gas pressure for 4 THz and 10 THz output bandwidths. (*c*) Spectral shape of the amplifier laser input needed to obtain 10-THz FWHM output spectrum. (Online version in colour.)

Figure 6 shows kinetics code simulations of the predicted energy output from a Nike-size e-beam-pumped ArF amplifier for two bandwidths (4 THz and 10 THz) and the intrinsic efficiency for gas pressures ranging from 0.8 to 2.0 amagats. The simulation indicates that one could obtain more than 16% intrinsic efficiency over a broad pressure range. The operating pressure is important in amplifier designs because it defines the stopping power of the laser gas, which determines the operating voltage for the e-beam diode. Our simulations indicate that one can obtain bandwidths up to 10 THz with ArF systems with little effect on the intrinsic efficiency if one arranges for a higher power input into the amplifier with the broader bandwidths. Figure 6*c* shows the spectral shaping of the input of the amplifier to obtain 10 THz FWHM output. Such spectral shaping with more power in the wings of the distribution can be obtained in the low energy front end of the laser system. This technique has been used on the Nike facility to increase the output bandwidth from the normal 1 THz FWHM to 3 THz FWHM. ArF has much broader bandwidth capability than KrF which allows the predicted bandwidth capability shown in figure 6 [30]. These laser kinetics predictions will be tested against experiment on the Electra ArF facility.

## 4. Development path to argon fluoride direct-drive inertial fusion energy power plants

**Figure 7.**
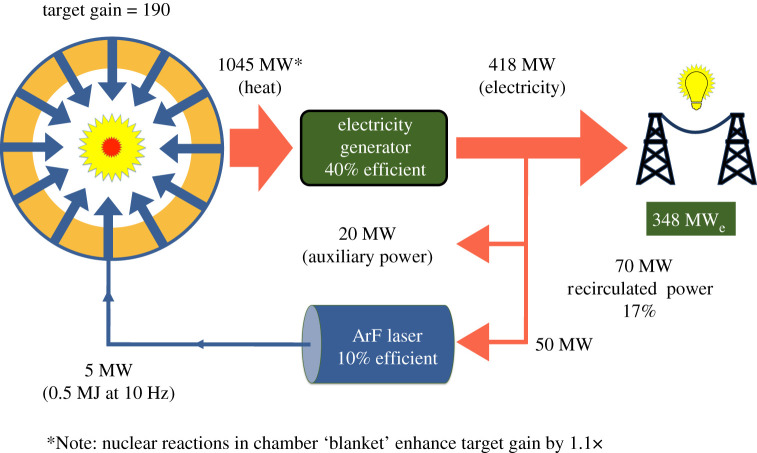
Power flow diagram for a power plant using a 0.5 MJ ArF laser and a high-gain direct-drive shock-ignited target. Most of the generated power (83%) is available for distribution to the grid due to the combination of a high-energy gain implosion (190) and a fairly efficient (10%) ArF laser driver. (Online version in colour.)

The greater than 16% intrinsic efficiency with an ArF driver would enable substantially higher electrical efficiency for delivering laser light to target than a KrF system. The projected electric efficiency is 7.2% for delivering laser light onto target with a KrF IFE system with 12% intrinsic efficiency [31]. Using the same analysis, an ArF system is projected to have 9.6% electrical efficiency with 16% intrinsic efficiency and 10.2% with 17% intrinsic efficiency. Figure 7 illustrates the potential to build modest size inertial fusion power plants with an ArF driver. Here a 0.5 MJ ArF driver provides a gain of 190 by means of a shock-ignited implosion. The gain estimate is 75% of that shown in figure 2 for shock ignition with 0.5 MJ. With a 10 pulse-per-second repetition rate the system generates 950 MW fusion power, which is amplified to 1045 MW thermal power by nuclear reactions in the lithium-containing blanket. For the parameters shown, 418 MW of electrical power is produced with 70 MW needed to power the ArF laser and other auxiliary systems, leaving 348 MW available to the grid. The potential for high gain at modest laser energy, and a 10% expected wall plug efficiency with ArF, could therefore enable much smaller, lower cost laser fusion power plants. The laser energy employed here is more than a factor of 6 smaller than the ‘SOMBRERO' inertial fusion power plant design study completed in 1993 that used a 3.4 MJ KrF laser [32]. The most cost-effective way to use such technology would likely be to construct power stations with multiple such power plant modules. That would reduce staffing and security cost and enable use of a single high-volume factory for low-cost target production.

**Figure 8.**
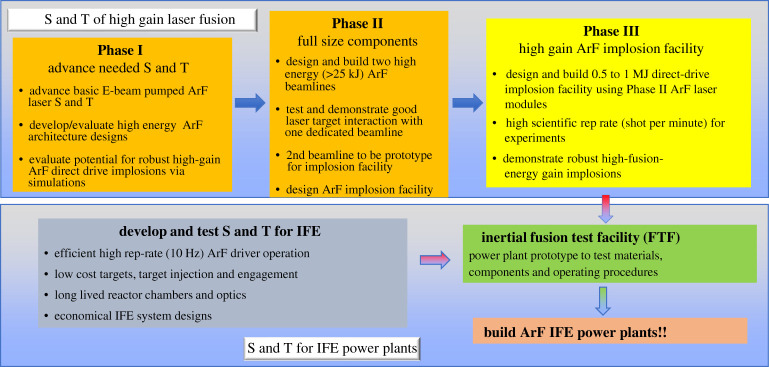
Phased development path to IFE power plants using an ArF driver that employs parallel target physics and IFE technology efforts. (Online version in colour.)

Figure 8 shows a phased development path to advance the science and technologies to the level needed to construct an inertial fusion power plant using an ArF laser driver. The path would involve development efforts to advance the physics of ArF laser target interactions and demonstrate the potential for high fusion gain. In parallel, the phased path would advance the technologies needed for a power plant, such as efficient high-energy, high-repetition rate ArF lasers and low-cost target fabrication. In Phase II of the physics path, one would build two ArF high-energy beam lines. One would be dedicated to testing and verifying the laser target interaction physics. The other would serve as a prototype module for a follow-on implosion facility capable of high-gain fusion. Energies of a few tens of kilojoules in the Phase II facility would allow one to conduct experiments with relevant laser produced plasma size and laser intensities to define LPI-safe operating conditions for pellet designs. In Phase III of the physics path, one would build and operate an ArF direct-drive implosion facility that would have the goal of advancing the target physics and demonstrating the high gains needed for energy. This could operate at a relatively high-repetition rate (compared to large glass laser facilities) of many shots per hour since ArF is a gas laser and is easier to cool.

The following step would be to construct a high-gain, high-repetition rate facility to develop the components, materials and operating procedures needed for a power plant. This inertial fusion test facility [33] would provide the detailed technical basis for designing and building power plants. This would require a broad range of technical advances in the ArF laser and other IFE technologies. For example, the period of operation between maintenance for the electron beam diode, pulsed power and optical components would need to be extended from the thousands of shots that is adequate for laser target interaction research lasers, to over 300 million if one wants to operate continuously for over a year at 10 pulses per second. A summary of technical challenges to direct-drive laser IFE systems is provided in reference [34].

ArF is a promising technology for achieving the high-gain inertial fusion implosions needed for energy [35]. The combination of broad bandwidth and short wavelength (193 nm) is expected to suppress LPI and thereby greatly broaden the design space available for laser direct-drive implosions. It may enable robust high-gain laser fusion with sub-megajoule energy. However, as a relatively undeveloped technology it will require a substantial investment to achieve the energy, repetition rate, precision and billion-shot class reliability required for a power plant. Our work so far indicates that there is no fundamental obstacle that would prevent an ArF direct-drive IFE system from meeting these requirements.

## Data accessibility

All data supporting the conclusions are provided within this document. Questions can be directed to the corresponding author.
